# Tacrine Derivatives in Neurological Disorders: Focus on Molecular Mechanisms and Neurotherapeutic Potential

**DOI:** 10.1155/2022/7252882

**Published:** 2022-08-18

**Authors:** Saikat Mitra, Maniza Muni, Nusrat Jahan Shawon, Rajib Das, Talha Bin Emran, Rohit Sharma, Deepak Chandran, Fahadul Islam, Md. Jamal Hossain, Sher Zaman Safi, Sherouk Hussein Sweilam

**Affiliations:** ^1^Department of Pharmacy, Faculty of Pharmacy, University of Dhaka, Dhaka 1000, Bangladesh; ^2^Department of Pharmacy, BGC Trust University Bangladesh, Chittagong 4381, Bangladesh; ^3^Department of Pharmacy, Faculty of Allied Health Sciences, Daffodil International University, Dhaka 1207, Bangladesh; ^4^Department of Rasa Shastra and Bhaishajya Kalpana, Faculty of Ayurveda, Institute of Medical Sciences, Banaras Hindu University, Varanasi, 221005 Uttar Pradesh, India; ^5^Department of Veterinary Sciences and Animal Husbandry, Amrita School of Agricultural Sciences, Amrita Vishwa Vidyapeetham University, Coimbatore, Tamil Nadu 642109, India; ^6^Department of Pharmacy, State University of Bangladesh, 77 Satmasjid Road, Dhanmondi, Dhaka 1205, Bangladesh; ^7^Faculty of Medicine, Bioscience and Nursing, MAHSA University, Jenjarom, 42610 Selangor, Malaysia; ^8^IRCBM, COMSATS University Islamabad, Lahore Campus, Lahore, Pakistan; ^9^Department of Pharmacognosy, College of Pharmacy, Prince Sattam Bin Abdulaziz University, Al-Kharj 11942, Saudi Arabia; ^10^Department of Pharmacognosy, Faculty of Pharmacy, Egyptian Russian University, Cairo-Suez Road, Badr City 11829, Egypt

## Abstract

Tacrine is a drug used in the treatment of Alzheimer's disease as a cognitive enhancer and inhibitor of the enzyme acetylcholinesterase (AChE). However, its clinical application has been restricted due to its poor therapeutic efficacy and high prevalence of detrimental effects. An attempt was made to understand the molecular mechanisms that underlie tacrine and its analogues influence over neurotherapeutic activity by focusing on modulation of neurogenesis, neuroinflammation, endoplasmic reticulum stress, apoptosis, and regulatory role in gene and protein expression, energy metabolism, Ca^2+^ homeostasis modulation, and osmotic regulation. Regardless of this, analogues of tacrine are considered as a model inhibitor of cholinesterase in the therapy of Alzheimer's disease. The variety both in structural make-up and biological functions of these substances is the main appeal for researchers' interest in them. A new paradigm for treating neurological diseases is presented in this review, which includes treatment strategies for Alzheimer's disease, as well as other neurological disorders like Parkinson's disease and the synthesis and biological properties of newly identified versatile tacrine analogues and hybrids. We have also shown that these analogues may have therapeutic promise in the treatment of neurological diseases in a variety of experimental systems.

## 1. Introduction

Neurological disorders are very complex and multifaceted, necessitating multitarget medications that can cause multiple subpathologies at once. These illnesses are characterized by progressive failure of certain neurotransmitter system and the improper functioning of neural networks in the brain [1, 2]. There have been a growing number of studies undertaken to discover changes linked with neurological disorders, including in neurotransmitters, transporters, receptors, and metabolizing enzymes. Through nicotinic and muscarinic cholinergic receptors on monoaminergic neurons, the cholinergic system can influence monoaminergic systems [3]. As a result, manipulating the cholinergic system can affect the signaling of other monoaminergic systems, and age-related changes in neurotransmitter systems can affect the response to such treatment techniques [4]. The use of small compounds to treat neurological disorders like Alzheimer's disease (AD) by modulating cholinergic and glutamatergic neurotransmissions has been suggested. A powerful acetylcholinesterase inhibitor (AChE), tacrine, was the first medicine approved by the US Food and Drug Administration for the treatment of AD [5]. It is a low-affinity N-methyl-D-aspartate receptor (NMDAR) antagonist. In addition to being an AChE inhibitor, tacrine has been revealed to have a variety of cholinergic actions, including boosting the synthesis and release of acetylcholine (ACh) and modulating the muscarinic and nicotinic receptors. It has also been shown that tacrine interacts with monoaminergic systems. A number of investigations have indicated that the memory-enhancing qualities of tacrine are more nuanced than previously thought. It has been shown to target a variety of pathways, including the thenitrinergic, gamma-aminobutyric acid (GABA)ergic, glutamatergic, and AChE pathways [6–10].

Although tacrine was the first drug approved for the treatment of AD, it was banned in 2013 by US Food and Drug Administration (FDA) due to its toxic effects on liver. After the prohibition, most of the research focused on finding safer analogues and of tacrine and its complex with other hepatoprotective agents to treat AD in a multitarget-directed ligand approach. Tacrine has effect against neurological disorders in a molecular basis [11].

This article explains the therapeutic effect of tacrine, its analogues and derivatives discovered in several preclinical studies in a variety of neurological disorders, especially Alzheimer's disease particularly from a pharmaceutical standpoint. Furthermore, this article includes an overview of tacrine and examines the molecular mechanistic role of tacrine effects against the neurological disorders.

## 2. Overview of Tacrine

Tacrine's pharmacological actions were initially characterized by Shaw and Bently in 1946 [7, 12]. It was first used in clinical practice to treat anesthetic-induced delirium and to enhance the muscle-relaxing effects of succinylcholine [13]. It was first thought that tacrine would help counteract the respiratory depression caused by morphine; however, it was later revealed that it also acted as an inhibitor of both AChE and butyrlcholine esterase (BChE) [9, 14]. In 1993, tacrine became the first medicine to be licensed for the treatment of Alzheimer's disease; however, its hepatotoxicity limited its usage [1]. Additional effects of tacrine have been discovered throughout time, including reduction of monoamine oxidase activity, inhibition of 5-hydroxytryptamine receptor and dopamine neuronal uptake, blockage of certain potassium ion channels, and interaction with muscarinic ACh receptors [15, 16].

Tacrine (9-amino-1,2,3,4-tetrahydroacridine, or THA) is an acridine containing three rings, but only a little substitution of an amino group in the fifth position is a white crystalline powder that dissolves in water, Tacrine's pKa value is 9.85, and it has a planar chemical structure [7]. Though it is totally ionized at pH 7, it is likely that the positive charge is spread out between the mesomeric structures [16]. Tacrine is quickly absorbed and has a bioavailability between 10% and 30%. Absorption can be reduced by as much as 40% when taken with food. Plasma proteins account for approximately 55% of the binding of tacrine. Its clinical half-life is approximately 3-6 hours after a single oral dose. Oral tacrine has a therapeutic dosage range of ten-fold, necessitating individualized dosing. The distribution volume appears to be 182 liters. During the last elimination phase, the plasma half-life is 2.5 hours on an average [7]. It has been reported incorrectly that 1-OH-tacrine, a pharmacologically active form of tacrine, is formed during metabolism. In reality, tacrine is broken down into up to seven distinct compounds during digestion, which include dextro-chiral (stereospecific) version of 1-OH-tacrine [14, 17].

Multiple modes of action exist for tacrine. Tacrine's primary mechanism of action in neurological disorders is as an acetylcholinesterase inhibitor that acts noncompetitively but selectively on the central nervous system (CNS). Moreover, CNS abnormalities extend beyond the cholinergic system and include serotonin neurotransmitter deficits, noradrenaline neurotransmitter deficits, and reduced vascular perfusion [18, 19]. Tacrine has some BChE selectivity over AChE. There are several other consequences on cholinergic system apart from enzyme inhibition: boost in production and secretion of ACh, activation of M1 subtype muscarinic receptors, suppression of M2 subtype muscarinic receptors and a raise in brain nicotinic receptors, and their potentiation at low and inhibition at high concentrations as well as a general potentiation of neuromuscular communication [20, 21]. Additionally, tacrine affects a wide range of biological targets, including neuromuscular junctions, potassium channels, sodium channels, and phosphorylation. Tacrine has been demonstrated to limit the absorption and increase 5-HT, noradrenaline, and GABA release; it also has an inhibitory action both monoamine oxidase (MAO)-A and MAO-B monoamine oxidase subtypes, and it suppresses brain histamine N-methyltransferase. As an example of this, tacrine has been demonstrated to reduce potassium-induced release of excitatory amino acids and block neuronal calcium channels in large dosages [21, 22].

In addition, tacrine was found to have a positive effect on ketamine side effects. It is mostly used to initiate and maintain anesthesia with the help of ketamine. Pseudo-hallucinations, delirium, and hypnagogic states were all common side effects of this medication's treatment. Postoperatively, tacrine has been shown to reverse psychotic episodes. It was discovered that tacrine's effects on the CNS were due to its interaction with adrenergic and cholinergic receptors [21, 23].

Pure anticholinesterase inhibitors like physostigmine and donepezil do not share two other peculiar properties of tacrine. An important benefit of tacrine in people with neurological disorders is that it greatly enhances cerebral blood flow. Decreased cerebral blood flow is linked to many neurological illnesses. Second, tacrine prevents the release of *β*-precursor protein from the intestinal tract. There are numerous metabolites that can be formed by metabolization of tacrine by cytochrome P450 IA2 system. In the early stages of tacrine administration, the hepatocellular response that is typical of many pharmacological drugs can occur. It is not known if tacrine and/or its metabolites are excreted in the bile or if they are transported via the enterohepatic system. The clearance of tacrine, however, does not appear to be affected by renal impairment [9, 15, 21].

## 3. Tacrine Derivatives

Tacrine (9-amino-1,2,3,4-tetrahydroacridine) acts by three times more effectively inhibiting butyrylcholinesterase (BChE) than acetylcholinesterase (AChE). When the carbocyclic ring was modified from a cyclopentyl (compound (1)) to a cycloheptyl ring (compound (2)), the efficiency against BChE activity enhanced approximately 4-fold, while the efficacy against AChE activity increased 2-fold. On the other hand, the anticholinesterase activity of the cyclooctyl derivative (compound (3)) was 100 times lower than that of tacrine, presumably owing to the carbocyclic ring's increased flexibility and bulk. The introduction of a methylene bridge in tacrine's cyclohexyl ring (compound (4)) leads to a reduction in anticholinesterase efficacy (IC_50_ against AChE: 0.24 *μ*M) that further significantly improved while the substituent bulk on the bridge is increased by three methyl groups compound (5) (IC_50_ against AChE:110.0 *μ*M). The inclusion of a fourth benzene ring led to a change in compounds with high AChE selectivity over BChE; just like in comparison to tacrine, compound (6) was extremely selective for AChE rather than BChE, with an IC_50_ of 0.35 *μ*M and 3.1 *μ*M against AChE and BChE, respectively. Against BChE (IC_50_: 6.7 *μ*M), tetracyclic compound (7) was 50 times more powerful than AChE (IC_50:_ 391.1 *μ*M) [24].

In addition to being more effective AChE inhibitors over tacrine, certain halogenated analogues have a greater affinity for AChE over BChE. Additional study has attempted at what emerges when a halogen atom is incorporated into the benzene ring of parent cyclopentyl analogue compound (1). The insertion of Cl at position 6, compound (10), elevated the strength by six fold; nevertheless, the 6F compound was equiactive with the parent cyclopentyl analogue. In aspects of selectivity against AChE being preferred over BChE (the B/A ratio), the 5Cl, 6Cl, 8Cl, 7F, and 8F derivatives, with B/A ratios of 4.1, 13, 3.8, 6.2, and 19, respectively, were the most selective compounds [25].

León et al. assessed the biological activity of new tacrine analogues possessing a heterocyclic ring moiety at position C-4, such as 2-thienyl, 3- or 4-pyridyl, and 2-(N-acetyl)-pyrrolyl groups. Among the pyrano[3,2-*e*]pyridines and pyrano[2,3-*b*]quinolines groups, the pyrrolyl possessing molecule compound (8) is by far the most effective, with an IC_50_ against AChE: 1.7 *μ*M, approximately 10-fold decline in levels of activity in comparison to tacrine. Compound (9) was the most powerful of the [1,8]naphthyridine derivatives, with an IC_50_ against AChE: 1.4 *μ*M [26].

Friedländer-type reactions of 2-aminopyridine-3-carbonitriles with 1-benzyl-4-piperidone or cyclohexanone have yielded new tacrine analogues. The biological analysis demonstrated that, in nanomolar range, certain of these compounds were effective AChE inhibitors, as well as selective inhibitors of BChE. Compound (11) is the shortest N2-alkyl substituted compound, and it performs better than the molecules with longer alkyl chain. This inhibitor has an identical AChE inhibition profile (IC_50_:14 nM) as tacrine, even though it is a weaker BChE inhibitor (IC_50_: 5.2 *μ*M). The insertion of an electron-withdrawing substituent like Cl at C-2 contributed in a substantial reduction in anticholinesterase activity. Furthermore, to enhance AChE selectivity, an N-benzyl was substituted for the methylene group at C-7, as demonstrated in two instances: (i) from compound (13) to compound (15) and (ii) from compound (14) to compound (16). Meanwhile, inhibitor A was 5.8 times more effective against EeAChE (AChE from *Electrophorus electricus*) in relative to compound B; both of these compounds possess a methoxy group at C-2. Nonetheless inhibitor C was 14 times less powerful than inhibitor D against EeAChE, and both compounds included a chlorine atom at C-2 [27].

Tacrine is covalently bonded to some other pharmacologically active compounds, including such as a M1 agonist xanomeline and (Z)-3-(4-chlorophenyl)-N´-((4-chlorophenyl)sulfonyl)-N-methyl-4-phenyl-4,5-dihydro-1*H*-pyrazole-1-carboximidamide known as an antagonist of CB1 receptor, which enables to consolidate tacrine's profound AChE inhibition with other pharmacological features [28]. The AChE inhibition of compound (17) is 1,000-fold broader than that of tacrine [29]. In respect to compound (17), compound (18) has a lower toxicity, yet will inhibit AChE and BChE in the same frequency as tacrine [28, 30].

Tang et al. created a variety of tacrine-oxoisoaporphine hybrids in their pursuit for more effective tacrine derivatives. In most instances, these compounds display significant AChE inhibitory potential, IC_50_ values being in nanomolar range, with the most impactful AChE inhibitor reported being compound (20) (IC_50_: 3.4 nM) [31].

Mao et al. discussed the design, synthesis, and assessment of a variety of o-amino benzylamine and o-hydroxyl tacrine hybrids, as well as o-hydroxyl benzylamine-(7-chlorotacrine) hybrids as multifunctional anti-AD drugs. Over these variants, compound (21) inhibits AChE (IC_50_: 0.55 nM) more effectively than tacrine [32].

**Figure 1:**
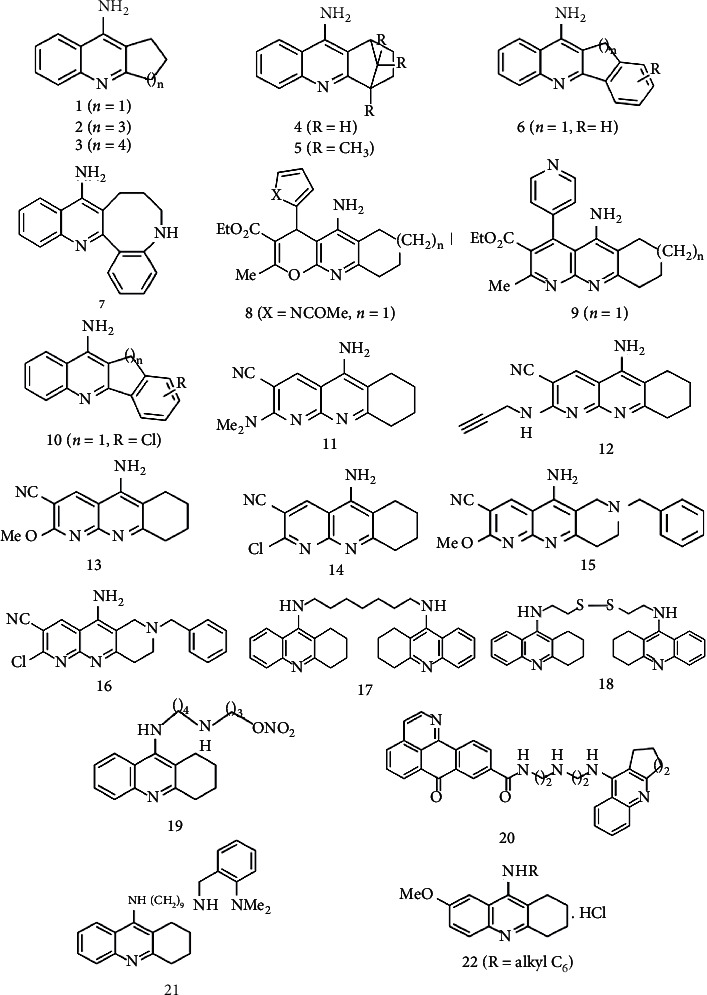
Illustration of compounds of tacrine analogues. The compound number denoted by (1) 9-Amino-2,3-dihydro-1H-cyclopenta[1,2-b]quinoline. (2) 11-Amino-2,3,4,5-tetrahydro-1H-cyclohepta[1,2-b]quinoline. (3) 12-Amino-1,2,3,4,5,6-hexahydrocycloocta[1,2-b]quinoline. (4) 9-Amino-1,4-methano-1,2,3,4-tetrahydroacridine. (5) 9-Amino-1,4-methano-1,2,3,4-tetrahydro-4,11,11-trimethylacridine. (6) 6-Amino-4,5-benzo-5H-cyclopenta[1,2-b]quinoline. (7) 9-Amino-5,6,7,8-tetrahydroquinolino[3,2-e]-1-benzazocine. (8) Ethyl 4-(1-acetyl-1H-pyrrol-2-yl)-5-amino-6,7,8,9- tetrahydro-2-methyl-4H-pyran[2,3-b]quinoline-3-carboxylate. (9) Ethyl 5-amino-6,7,8,9-tetrahydro-2-methyl-4-(4- pyridyl)-benzo[b][1–8]naphthyridine-3-carboxylate. (10) 9-amino-6-chloro-2,3-dihydro-[1H] cyclopenta [1,2-b]-quinoline. (11) 5-Amino-2-(dimethylamino)-6,7,8,9-tetrahydrobenzo[1,8-b]-naphthyridine-3-carbonitrile. (12) 5-Amino-2-(prop-2-yn-1-ylamino)-6,7,8,9-tetrahydrobenzo[1,8-b]-naphthyridine-3-carbonitrile. (13) 5-Amino-2-(methyloxy)-6,7,8,9-tetrahydrobenzo[1,8-b]-naphthyridine-3-carbonitrile. (14) 5-Amino-2-chloro-6,7,8,9-tetrahydrobenzo[1,8-b]- naphthyridine-3-carbonitrile. (15) 5-Amino-7-benzyl-2-methoxy-6,7,8,9- tetrahydropyrido[2,3-b][1,6]naphthyridine-3-carbonitrile. (16) 5-Amino-7-benzyl-2-chloro-6,7,8,9- tetrahydropyrido[2,3-b][1,6]naphthyridin-3-carbonitrile. (17) Bis(7)tacrine dimer. (18) Cystamine-tacrine dimer. (19) Nontoxic tacrine-organic nitrates, compound E (as the name wasn't found in the paper). (20) N-(7-Oxo-7H-dibenzo[de,h]quinolin-9-yl)-3-((2-((1,2,3,4-tetrahydroacridin-9-yl)amino)ethyl)amino)propanamide. (21) N1-(2-(Dimethylamino)benzyl)-N9-(1,2,3,4-tetrahydroacridin-9-yl)nonane-1,9-diamine. (22) N-alkyl-7-methoxytacrine.

**Figure 2:**
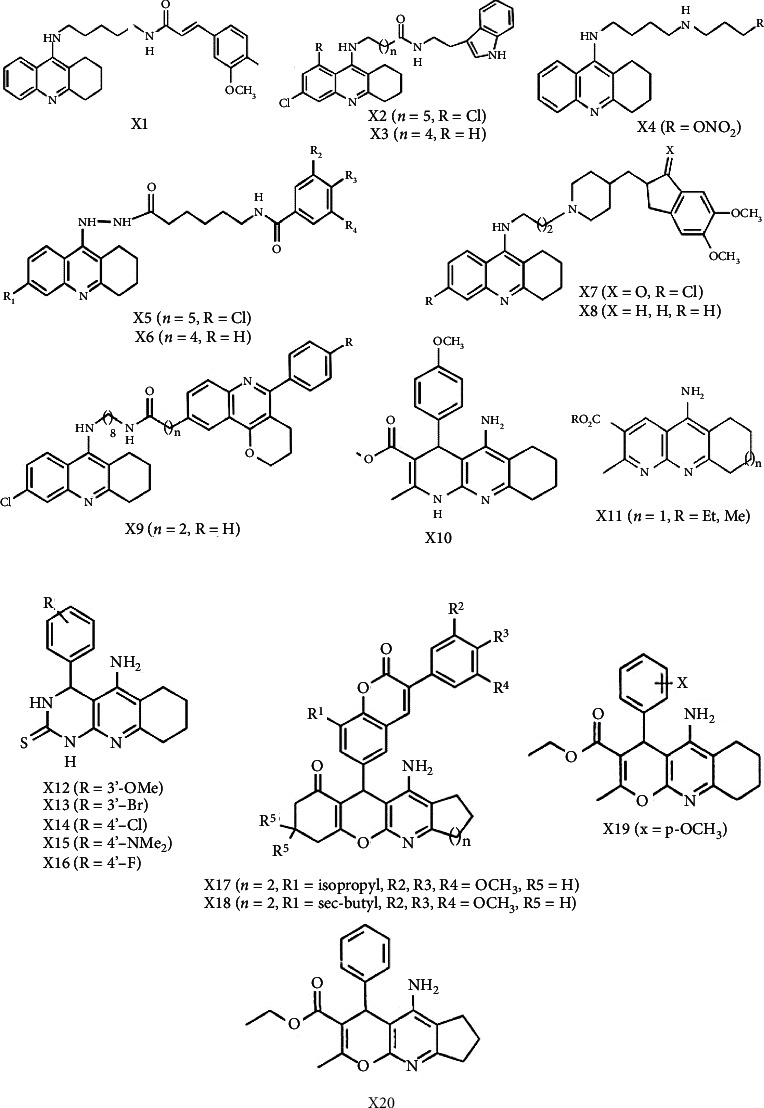
Illustration of compounds for neuropharmacological potential of tacrine hybrids.

7-MEOTA (9-amino-7-methoxy-1,2,3,4-tetrahydroacridine), a new AChE inhibitor, was shown to be exempt of several toxicities particularly in comparison to tacrine (Figures 1 and 2). Korabecny et al. generated a number of 7-MEOTA analogues by alkylating the aromatic amino moiety of this new AChE inhibitor, with intriguing findings. C5–C9 chains outperformed their parent molecule 7-MEOTA and also in specific instance of inhibition of hAChE (human recombinant AChE), compound with a hexyl chain being the most effective (IC_50_: 0.10 *μ*M) [33].

## 4. Molecular Basis of Tacrine Derivatives' Action against Neurological Disorders

### 4.1. Modulation of Neurogenesis

Animal and human brains undergo a process known as neurogenesis, in which new nerve cells are continuously created throughout the life span of the organism. They are capable of differentiating into functional cells of the central nervous system and integrating into existing neural circuits in the brain after they have been born [6, 34]. Therapy for Alzheimer's disease may be possible if neuronal regeneration (neurogenesis) is targeted in the hippocampus [35]. Subventricular zone (SVZ) and dentate gyrus (DG) of the hippocampus are two regions of the mammalian brain where neurogenesis continues into adulthood. When NPCs are multiplied in many locations, new neurons and glial cells can be generated in the brain [8, 36, 37]. With the help of the bromodeoxyurine (BrdU)-labeling paradigm, Jin et al. determined the effects of tacrine on neurogenesis in adult mice. According to the findings, it increases neurogenesis in the DG and SVZ by 26–45%, although it has no effect on BrdU labeling in the DG [35]. In order to study the effects of compound (17), an acetylcholinesterase (AChE) inhibitor, on cognitive performance, neuronal apoptosis, and neurogenesis in the hippocampal region of adult male Sprague-Dawley rats, was permanently ligated of the bilateral common carotid arteries [38]. The neuroprotective effects of tacrine and its analogues have been proven in several investigations, including those by Li et al. and Luo et al. Tacrine and its analogues antagonize glutamate excitotoxicity and reduce neuronal nitric oxide synthase activity [39, 40]. Farkas et al. discovered that administering compound (17) to a chronic cerebral ischemia rat corrected the impairments in spatial learning and memory that had previously been seen. In the hippocampus, the neuroprotective impact of compound (17) may be exerted through the promotion of neurogenesis in the DG and the inhibition of apoptosis in the CA1 area, respectively. They observed that this impact could potentially be due to activation of cholinergic receptors that are expressed on neural progenitors and that these receptors in turn may promote neurogenic factors [41].

### 4.2. Modulation of Neuroinflammation

The recognition of the role played by inflammation in the progression of a wide range of diseases has prompted innovative efforts to discover therapeutic approaches to reduce inflammation. Cholinergic deficiency and neuroinflammation are critical factors in both acute and long-term CNS disorders [2, 21, 42]. Glial cell activation (astrocytes and microglia) is a frequent feature of many neurodegenerative illnesses, and it is largely responsible for this process. Glial cells activated during brain inflammation may produce proinflammatory mediators, which have been linked to the neuropathology that produces cognitive impairments in neurological diseases [43, 44]. In neurodegenerative illnesses, the balance between proinflammatory and anti-inflammatory cytokines also has an impact on the course of the disease [15, 45]. According to Zhang et al., hippocampal injection of a tacrine analogue known as 4-(bis(pyridin-2-ylmethyl)amino)-N-(3- ((1,2,3,4-tetrahydroacridin-9-yl) amino)propyl)butanamide could effectively attenuate A*β*1-42 oligomers-induced cognitive dysfunction by activating the CREB/BDNF signaling pathway, decreasing tau phosphorylation [46]. According to the findings of a study conducted by Tyagi et al., tacrine may have an inhibitory effect on lipopolysaccharide (LPS-)-induced neuroinflammation in adult Swiss albino mice, as evidenced by a decrease in the high levels of IL-2 in the brain [42]. Being AChE inhibitors, tacrine and its analogues work by increasing the availability of acetylcholine in central cholinergic synapses and are hence considered as the most promising medications now available for the treatment of neurological diseases like AD [47, 48]. As per Jeřábek et al., tacrine-resveratrol-fused hybrids have the potential to be exploited as multitargeted ligands against neurological diseases especially AD due to their fascinating anti-inflammatory and immunomodulatory capabilities in neuronal and glial AD cell models [49]. Moreover, in a cell-based experiment conducted by Alfadly et al., the antineuroinflammatory and neuroprotective characteristics of tacrine were demonstrated by a reduction in the expression of IL-1*β* and TNF-*α*, as well as the formation of ROS, in LPS-challenged PC12 cells following treatment with the chosen inhibitors [50]. Tacrine, as discovered by Xu and Xu, alleviated thermal hyperalgesia and mechanical allodynia following sciatic nerve chronic construction injury while also restoring functional morphological damage in rats. Besides that, tacrine inhibited the growth and activation of glia and decreased IL-1, IL-6, and TNF-*α* level in the blood. Tacrine also had an anti-inflammatory effect on the JAK2/STAT3 signaling pathway, which is involved in neuroinflammatory processes. These findings suggest that tacrine may be a good option for use as an analgesic drug in the treatment of neuropathic pain [51].

### 4.3. Modulation of Endoplasmic Reticulum Stress

Inhibition of DNA synthesis by tacrine results in functional and structural alterations in the liver's endoplasmic reticulum, ribosome, and mitochondria. As an acetylcholinesterase inhibitor (AChEI), tacrine has the potential to interfere with the normal trafficking of AChE in the endoplasmic reticulum (ER) [48, 52]. Tacrine exposure in neuronal cells with AChE (e.g., neurons) resulted in accumulation of misfolded AChE. Due to ER stress and the unfolded protein response's downstream signaling cascade, this misfolded enzyme is incapable of moving to its target, resulting in neuronal death via apoptosis [7, 53]. ER-mitochondrial collaboration enhanced mitochondrial membrane potential loss when the stress level was too high. After a while, the tacrine-exposed cells were unable to maintain homeostasis any longer and eventually died. Tacrine promoted ER stress and apoptosis, which were inversely related to the concentration of AChE [9]. By causing ER stress in neurons, other AChEIs (rivastigmine and bis(3)-cognitin) could create the same problem as tacrine. Tacrine and other AChEIs interfere with AChEe's normal transport across the ER. Once neurons and other cells have been damaged, they die. AChEIs for the treatment of neurological disorders like AD may benefit from these findings, which can be used to guide the development of new drugs [10, 39, 54, 55]. Using cultured neuronal cells, Liu et al. found that tacrine disrupts oligomeric acetylcholinesterase's ability to properly assemble, leading to endoplasmic reticulum–stressed death [54].

### 4.4. Modulation of Apoptosis

Apoptosis is a process in which cells actively participate in their own death. The regulation of apoptotic cell death can be regulated by a number of genes like bcl-2 family [56, 57]. Although the exact origin and mechanism of neuron death in neurological disorders like AD are still unknown, research suggests that oxidative stress may play a role. During neurodegeneration, reactive oxygen species (ROS) have been proposed to play a significant role in oxidative damage, which can be created by the cell lysis, by an excessive amount of free transition metals, or by an oxidative burst [7, 17]. Alzheimer's disease patients' brains appear to degenerate by an apoptotic process, including the presence of damaged DNA, nuclear apoptotic bodies, and other apoptosis-related markers in postmortem tissue samples. As a result of these findings, it is possible that apoptosis can be prevented or delayed by using ROS-inhibiting therapies, which may be a viable treatment option for the disease [58, 59].

Wang et al. reported that tacrine protects against H_2_O_2_-induced apoptosis, presumably by preventing the production of proapoptotic genes such as p53 and bax in the cells [58]. A tacrine analogue called as tacrine(2)–ferulic acid was found to inhibit 6-hydroxydopamine-induced apoptosis in rat pheochromocytoma (PC12) cells by activating the phosphoinositide 3-kinase (PI3-K)/Akt signaling pathway, according to the findings of Zhang et al. [57]. Gao et al. (2013 and 2014) reported that tacrine causes apoptosis, thereby imposing cytotoxicity in HepG2 (American Type Culture Collection HB-8065) cells via a lysosome- and mitochondria-dependent mechanism and increased intracellular ROS production [17, 60]. Apoptosis has been linked to oxidative stress, and prior research has shown that tacrine, huperzine A, and donepezil protect against exogenous *β*-amyloid-induced damage and ameliorate redox disequilibrium in aged rats [56, 57, 61, 62]. Tacrine may be a helpful neuroprotective medication that lowers oxidative stress-induced cell death, which may complement its AChE inhibitory capabilities for the treatment of neurological illnesses. One of the hallmarks of apoptosis is oxidative stress, which has been linked to a wide range of cell death mechanisms and which in turn is triggered by ROS. YCG063 was tested as a ROS inhibitor by Gao et al., to see if it affected tacrine-induced apoptosis. The results show that YCG063 greatly suppresses tacrine-induced apoptosis, implying that tacrine-induced apoptosis is directly linked to ROS generation [17]. Apoptosis is triggered by tacrine, and ROS is a key player in this process. To put it another way, oxidative stress can lead to mitochondrial permeability transition (MPT) if ROS levels are greater than the cell's capacity to deal with them, which in turn increases ROS levels and the stress [17, 60, 63].

AChE activation by tacrine and its analogues might also possibly play a crucial role in apoptosis, as suggested by various research studies [7, 26, 48, 64, 65]. Apoptosis was increased in retinal cells from chick embryos transfected with AChE sense vectors, and highly purified AChE proteins have been demonstrated to be harmful to cells via the apoptotic mechanism. These findings suggest that AChE is involved not only in mammalian organ development but also in regulating how well it can respond to external stimuli [57, 58]. These noncholinergic functions of AChE may be crucial in neurological disorders, and tacrine may be able to influence them positively [4, 7].

### 4.5. Role in Energy Metabolism

Alterations in cellular energy metabolism and mitochondrial dysfunction are two further aspects that contribute to the pathophysiology of neurological disorders [5, 66]. Multitarget-directed ligands combine cholinesterase inhibition with antioxidant capabilities to favorably alter neuronal energy metabolism and mitochondrial function to reduce illness symptoms [67–69]. The effect of ChE inhibitors (tacrine and 7-methoxytacrine) on the activity of mitochondrial complex I in the brain of a pig was investigated by Korábečný et al. and Hroudová et al., in mitochondria from the brain of the animal [70, 71]. Tacrine dramatically reduced the activity of mitochondrial complex I, which may result in tacrine-induced deleterious consequences associated with imbalances of electron transport chain [7, 72]. A disruption in the activity of complex I, which is critical in the regulation of oxidative phosphorylation, could result in impairment of cellular energy metabolism and, as a result, abnormalities in neuronal activity. As a result of inhibition of oxidative phosphorylation, glycolytic energy generation can become more prominent in tissues with high-energy requirements, such as the brain, which is especially true for the brain [71, 73]. Berson et al. reported that the weak base tacrine has a protonophoric action in mitochondria due to its high proton concentration. When the cell's energy expenditure increases without a concurrent increase in ATP production, the result is cell malfunction at low dosages and cell death at high levels, according to the literature. These mitochondrial effects are observable in cells from either rats or humans who have been treated to tacrine at clinically relevant concentrations or dosages [74].

### 4.6. Regulatory Role in Gene and Protein Expression

Tacrine regulates Kv2.1 channel gene expression and cell proliferation, according to Hu et al. [34]. Transcription factors have been shown to influence the expression of proapoptotic Bcl-2 and antiapoptotic Bax genes, respectively, in response to the p53 tumor suppressor gene [75]. In both developed and undifferentiated human neuroblastoma cell lines, Lahiri et al. found that tacrine altered the secretion of the *β*-amyloid precursor protein. Fu et al. found that compound (17) has been shown to be effective in protecting primary cultured astrocytes, pheochromocytoma cells, and neurons from hydrogen peroxide-induced apoptosis as well as against amyloid beta protein and glutamate-induced apoptosis. Tacrine derivatives accelerate human glioma SF295 cell death and change glioblastoma-related proteins such as p53, *β*-catenin, MAP2c, Iba-1, Olig-2, HLA-DR, and IDH1proteins related to disease development [76]. ChAT and M3 and M5 muscarinic receptor subtypes downregulation in chronic hypoperfusion-induced cognitive deficits can be alleviated by tacrine therapy, according to the findings of Zhao et al. [77]. By protecting neurons against amyloid *β* protein-induced cell death and apoptosis, tacrine may serve as more than just an AChEI. It may also stimulate antiapoptotic gene expression by lowering oxygen-free radicals during neuronal injury [78]. Alfirevic et al. reported that tacrine-induced transaminitis was linked to genetic variations in ABCB4, which encodes the MDR3 phosphatidylcholine transporter. Tacrine does not appear to be a substrate or inhibitor of MDR3 [79].

### 4.7. Role in Ca^2+^ Homeostasis Modulation

Cell death can be controlled by calcium, which is a second messenger in the body. It has even been proposed that the final common mechanism for all types of cell death is Ca^2+^ overload [80]. The imbalance of Ca^2+^ in the body is now recognized as a common factor in the pathophysiology of Alzheimer's disease [81]. Ca2+ has recently been linked to the toxicity and buildup of amyloid protein, which, in turn, shows collaboration with tau protein [82]. Since nimodipine, a well-known calcium channel blocker, and tacrine, a well-studied calcium channel blocker, have been combined structurally, Marco-Contelles et al. have produced tacripyrines, a new therapeutic candidate (lead drug candidate RL2/101) [83]. It has been demonstrated that RL2/101 interacts with the peripheral anionic site of AChE as well as the L-type of Ca^2+^ channels concomitantly. Several studies have shown that tacrine affects the homeostasis of Ca^2+^. Tacrine has been shown to inhibit high-threshold calcium currents by Kelly et al. [82] and Lermontova et al. [84]; however, their investigation required significantly larger concentrations of tacrine, and they were unable to identify the channels involved. Tacrine had no effect on calcium levels in synaptosomes, according to Gibson et al. [85] and Marco-Contelles et al. [83]. Doležal et al. [86] showed that tacrine attenuates the influx of calcium by blocking L-type and N-type calcium channels in cholinergic SN56 neuronal cell lines. This inhibitory action is not a consequence of the AChE activity of tacrine. An important consideration in determining tacrine's therapeutic potential is whether or not low micromolar concentrations of the drug interfere with calcium-dependent processes. Tacrine-dihydropyridine hybrids have a great neuroprotective profile and a modest blocking action on L-type voltage-dependent calcium channels due to the moderation of [Ca^2+^] elevation triggered by K^+^ depolarization. Chioua et al. found that blocking the entry of Ca^2+^ ions through L-type voltage-gated calcium channels by tacrine is a valuable strategy to prevent neuronal damage in neurological disorders like AD [73]. Tacrine treatment in male Crl:CD mice significantly increased the mitochondria's vulnerability to calcium-induced mitochondrial permeability transition, according to Mansouri et al. [59].

### 4.8. Osmotic Regulation

Tacrine is structurally similar to 9-aminoacridine, which is an open Na^+^ channel blocker, as well as 4-aminopyridine, which is a K^+^ channel blocker. Several investigations have demonstrated that tacrine has a direct effect on Na^+^, K^+^, and Ca^2+^ channels [7]. Tacrine reduces Na+ currents at a rate that is much larger than K+ currents, according to Adem [87], who performed voltage clamp tests on myelinated axons. It was recently reported that THA-induced action potential lengthening is caused by a modified Na+ current inactivation and delayed K+ current activation, which was proven using a similar preparation. Neuronal firing patterns and direct transmitter release are theorized to be affected by an action potential prolongation in some systems [9]. They further suggest that tacrine preferentially inhibits Na+ channels in the open state, whereas tacrine largely inhibits K+ channels the closed state, further supporting the previous findings. After an initial period of inhibition, ACh is administered to pyramidal neurons in the cerebral cortex, resulting in a progressive increase in activity followed by another period of inhibition. Muscarinic receptors, which may be selectively inhibited by pirenzepine, appear to be responsible for the delayed excitatory response, and these responses are mediated through voltage-dependent K+ conductance, which decreases with voltage [22, 44, 70]. Tacrine (and ACh in the synapse) act on M1 muscarinic receptors, which are blocked by pirenzepine, preventing the release of ACh from the slices, as previously explained. Pirenzepine blocks the release of ACh from the slices. A reduction in voltage-dependent K+ conductances may occur as a result of the impact of tacrine (and ACh) on muscarinic M1 receptors, which would lead to an increase in the release of Ach [3, 21]. However, a direct action of tacrine on K+ channels may boost ACh release, which cannot be totally ruled out. Thus, tacrine's direct and/or indirect effects on the different ion channels may contribute to its therapeutic success in the treatment of Alzheimer's disease [88, 89]. A low osmotic pressure makes tacrine unsuitable for use as an osmotic dose form in the gastro-intestinal tract [7, 87].

## 5. Therapeutic Potential of Tacrine Derivatives against Neurological Disorders

By blocking the enzyme acetylcholinesterase, tacrine prevents acetylcholine from being degraded, therefore augmenting acetylcholine levels. The therapeutic utilization of tacrine for AD therapy has been halted due to its toxicity to the liver, incurred by an elevation in liver transaminase and a decline in liver albumin. Meanwhile, medications that regulate a single target may not be therapeutically successful in multifactorial disorders like Alzheimer's. As a corollary, a number of medicines acting on various targets particular to multifactorial diseases have been derived. Multitargeted-directed ligands (MTDLs) are the term for those kinds of novel medications.

### 5.1. Alzheimer's Disease

There are millions of older people throughout the world who are suffering with Alzheimer's disease (AD), a debilitating and incurable neurological illness. The treatment options available currently only achieve some short-term relief from the cognitive symptoms. The major mechanisms of this disease include accumulation of amyloid beta (A*β*) proteins forming neurofibrillary tangles, loss of cholinergic activity, elevation of oxidative stress, and disruption of homeostasis of transition metal ions [90]. Tacrine was the first approved drug against Alzheimer's disease which was approved by the FDA in 1993. It worked as an inhibitor of cholinesterase [91]. But due to its liver toxicity, it was discontinued in the USA in 2013. Since then, tacrine has been used as a lead compound and combined with numerous ligands to create multitarget-directed ligands (MTDLs) or drugs to combat against the multifactorial aspects of AD.

**Figure 3:**
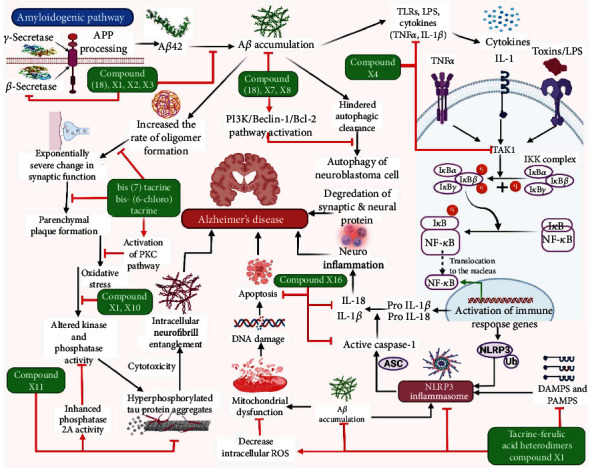
Illustration representing the site of action of different tacrine derivatives in Alzheimer's disease.

Homodimer of tacrine known as compound (17) is a potential lead compound of novel MTDLs. The heptamethylene linker of compound (17) was replaced with the structure of cystamine, forming compound (18) (Figure 3). This dimer exhibited a lower toxicity and greater ability to act as cholinesterase inhibitor, against both AchE (acetylcholinesterase) and BchE (butyrylcholinesterase). It is also able to inhibit beta-amyloid aggregation and displays a neuroprotective action against oxidative injury induced by H_2_O_2_ on SH-SY5Y cell line. According to research, the chemical (18) exerts its neuroprotective effects through activating kinase 1 and 2 (ERK1/2) and the Akt/protein kinase B (PKB) pathways [92].

By synthesizing new multifunctional tacrine-trolox hybrids, which have both ChE inhibitory and potent antioxidant activities akin to the mother molecule, trolox, Xie, Lan et al. were able to create compounds with both of these features. Compound 6d was shown to be the most effective inhibitor of AChE in male Sprague-Dawley (SD) rats (IC50 value of 9.8 nM for eeAChE and 23.5 nM for hAChE). A powerful inhibitor of BuChE was also found (IC50 value of 22.2 nM for eqBuChE and 20.5 nM for hBuChE). Studies in molecular modeling and kinetics reveal that 6d is a mixed-nature inhibitor that binds to both the CAS and PAS regions of AChE concurrently. The hepatotoxicity of 6d was shown to be substantially lower than that of tacrine in in vivo tests. A neuroprotective impact as well as excellent BBB permeability was also demonstrated. Overall, 6d can be considered a multifunctional drug for the treatment of Alzheimer's disease (AD) [93].

Xie, Wang, et al. designed, synthesized, and tested another class of new multitarget compounds against Alzheimer's disease: tacrine-coumarin hybrids. Most of the substances studied have robust AChE and BuChE inhibitory action, as well as clearly selective MAO-B inhibitory activity. Inhibition of AChE and BuChE by 14c (IC50 values for eeAChE and 16.11 nM and 33.63 nM, respectively) and BuChE was observed in the produced compounds (IC50 values of 80.72 nM for eqBuChE and 112.72 nM for hBuChE). As a competitive inhibitor of MAO-B, 14c is an effective anti-AD multitargeted drug with increased CNS penetration and reduced cell toxicity [94].

In this study, Zha et al. developed and synthesized twenty-six new tacrine–benzofuran hybrids and investigated their antiefficacy Alzheimer's in vitro on recombinant human AChE (hAChE) and BChE (hBChE) from human serum. Inhibiting human acetylcholinesterase at a subnanomolar level (IC50 = 0.86 nM) and suppressing both hBACE-1 activity (IC50 = 1.35 M) and aggregation of -amyloid (IC50 = 0.86 nM) (hAChE- and self-induced, 61.3 percent and 58.4 percent, respectively). Tests on scopolamine-treated ICR mice found that 2e significantly improved their performance and had no hepatotoxic effects [95].

Experiments using in-silico docking modeling chose a linker that best suited the bimodal drug's interaction with the active site of acetylcholinesterase enzyme (AChE). It was shown that compounds 9d and 9l had the highest results for AChE inhibition and for preventing superoxide formation as well as A-induced cellular toxicity, respectively, when compared to all other compounds tested in this study [96].

Bis(7)tacrine, which has a heptamethylene connection between two tacrine units, seems to be the most intriguing tacrine homodimer. It has a greater IC_50_ value of 1.5 nM for AChE inhibition as well as a superior pharmacological characteristic than tacrine. The potential of bis(7)tacrine to operate on several AD targets was linked to its neuroprotective action.

Designing novel anti-AD-modifying drugs entails inhibiting BACE-1, an enzyme concerned for *β*-amyloid peptide (A*β*) formation. Apparently, bis(7)tacrine has been reported to suppress BACE-1 with an IC_50_ of 7.5 *μ*M. In Neuro2a APPswe cells, bis(7)tacrine reduced the quantities of both intracellular and secreted A*β* without affecting APP expression (Figure 3). BiS(7)tacrine, like other multifunctional tacrine compounds, reduced AChE-induced A*β* aggregation. The multifunctional nature of bis(7)tacrine for AD therapy was further emphasized by the fact that it blocked both KV1.2-encoded potassium channels and native delayed rectifier potassium channels like tacrine. Furthermore, bis(7)tacrine is an antagonist of the *γ*- aminobutyric acid type A (GABA_A_) receptor, having an IC_50_ of 6.28 *μ*M. This observation leads to the inference that AChEIs with considerable GABA_A_ receptor antagonism would be more effective for treating AD than simple AChE inhibition alone [7].

Heptylene-linked bis-(6-chloro) tacrine, another homodimeric congener, has a substantially higher potency, with an IC_50_ of 0.07 nM. Heptylene-linked bis-(6-chloro)tacrine was shown to be 3000 times more effective than tacrine and interestingly 3 times more potent than bis(7)tacrine in inhibiting rat AChE. The inclusion of a halogen at the 6-position of such homodimeric tacrines resulted in a remarkable enhancement in AChE inhibitory efficacy with AChE/BChE selectivity [97].

Tacrine-ferulic acid heterodimers are generated by connecting ferulic acid and tacrine with a polymethylene diamine-type spacer. The antioxidant characteristics of ferulic acid, along with its neuroprotective efficacy against A*β*_42_ toxicity in both in vitro and in vivo experiments, prompted the selection of this compound. It was found that compound X1 was simultaneously noncompetitive and reversible AChEI and implies that the enzyme's PAS may interact with it. In an oxygen radical absorbance capacity (ORAC) analysis, compound X1 demonstrated a high ability to lessen the quantity of reactive oxygen species (ROS), like one might predict given the inclusion of the ferulic moiety [98–101].

Hybrids of tacrine and melatonin were also developed as AD therapy drugs with several functions. Melatonin was considered owing to its potential to neutralize a wide range of ROS in the body. It moreover promotes the activity of endogenous antioxidant enzymes, protects against A*β*, and suppresses neurofilament hyperphosphorylation. The most powerful AChEIs in this series, with IC_50_ values in the subnanomolar range and antioxidant activity in an ORAC experiment, were X2 and X3. Comparing to propidium, compound X3 suppressed the self-promoted aggregation of the A*β* (63% at 10 M). It has a 16.2% neuroprotection against A*β*_25-35_ at 1 *μ*M as well as cell viability of 100% at 1 *μ*M and 80% at 10 *μ*M. Furthermore, in neuroblastoma cells, X3 reduced rotenone-induced oxidative damage by 30% at 3 *μ*M. Rotenone acts as an apoptosis inducer and suppresses mitochondrial complex I specifically [102, 103].

NO enhances blood flow and minimizes inflammatory responses, while both of which may be pharmacologically advantageous for Alzheimer's patients. On this premise, tacrine was coupled to a NO donor unit using a suitable alkylenediamine spacer to generate novel tacrine heterodimers. In vitro study using PGF2-precontracted porcine pulmonary arteries demonstrates NO donor unit's vasorelaxing properties. X4 was the most powerful of the heterodimers studied, with an EC50 of 8.80 *μ*M, whereas isosorbide dinitrate as a positive control had an EC50 of 0.42 *μ*M and tacrine as a negative control had an EC50 of 97.3 *μ*M. Unlike tacrine, X4 seemed to have no consequence of hepatotoxicity, since albumin, lactate dehydrogenase (LDH), and aspartate aminotransferase (ASAT) levels were unaffected by this heterodimer. Interestingly, in behavioral tests, X4 showed a significantly greater capacity to manage scopolamine-induced memory impairment compared to tacrine [104].

In order to target both the AChE and the muscarinic M_2_ receptor allosteric site, tacrine-gallamine heterodimers were generated. This is because tacrine additionally functions as an allosteric modulator for muscarinic receptors, while gallamine blocks the AChE PAS and acts as selective allosteric modulator for muscarinic M_2_ receptor subtypes. X5 was the most powerful AChEI of the heterodimers studied in this series, with an IC_50_ of 5.44 nM. Tacrine-gallamine heterodimers slowed [^3^H]N-methyl scopolamine dissociation, particularly X6 with an EC50 value of 0.9 nM outperforms tacrine and gallamine by 4800 and 100 times, respectively. The ability of X6 to bind concurrently with the core and peripheral portions of the muscarinic M_2_ receptor allosteric site might explain this augmentation in efficiency [105, 106].

Tacrine-donepezil heterodimers were generated by substituting donepezil's benzyl moiety with tacrine connected with a polymethylene spacer. With an IC_50_ value of 0.27 nM, X7 was found to be the most powerful AChEI. The AChE-induced A*β* aggregation was suppressed by X7 and X8, by 46.1% and 65.9% at 100 *μ*M, hence demonstrating their interaction with the AChE PAS and their possible involvement in modulating AD pathology [107].

Among tacrine-propidium heterodimers, one of the balanced and intriguing compounds is X9. It decreased AChE-induced Ab aggregation by 45.7% at 100 *μ*M and modestly inhibited A*β*_1-42_ aggregation, according to molecular modeling studies. Furthermore, this molecule blocked BACE-1 and was demonstrated to pass the blood-brain barrier, allowing it to approach its therapeutic targets in the CNS [7].

Tacripyrines, a new class of hybrid compounds, were developed by combining the calcium antagonist 1,4-dihydropyridine (DHP) skeleton with the tacrine in order to suppress AChE while also suspending L-type Ca^2+^ channels. Cell death happens as a consequence of excess Ca^2+^ influx via L-type Ca^2+^ channels. In Alzheimer's disease, Ca^2+^ dysfunction promotes tau hyperphosphorylation and A*β* production [7]. Compound X10 was found to be the most promising because of its ability to inhibit hAChE with an IC_50_ of 105 nM, as well as 34.9% moderate inhibition of self-aggregation of A*β* and 30.7% inhibition of AChE-induced A*β* aggregation [108]. Furthermore, it is enriched with the capability to block Ca^2+^ (albeit lower than nimodipine) and to protect the brain from oxidative stress. It also inhibits AChE-induced Ab aggregation and may penetrate the BBB, as shown by an in vitro BBB permeability experiment [7]. Furthermore, X10 was revealed to be as effective as 3-{4-[(benzylmethylamino)methyl]phenyl}-6,7-dimethoxy-2*H*-2-chromenone (AP2238) and even more powerful than donepezil [108].

The PyridoTacrine family, which possesses the 1,8-naphthyridine motif, exhibits increased ChE inhibition and reduced hepatotoxicity while losing VGCC blocking efficacy. X11 is an MTDL for Alzheimer's disease since it offers pharmacological activity against 2 different therapeutic targets related to neurodegeneration: ChE and PP2A. In animal models of AD and stroke, this compound demonstrated its therapeutic potential. In fact, when compared to BuChE, tacrine derivative X11 is a more powerful and selective AChEI. By activating PP2A, it may be shielding against glutamate-induced excitotoxicity. PP2A inhibition causes tau hyperphosphorylation, which culminates in the generation of neurofibrillary tangles inside neurons, one of the most prominent indications of Alzheimer's disease. PyrTac X11 lowers scopolamine-induced memory impairment, which might be encouraging outcomes for therapeutic application [2].

A tacripyrimidine series was generated by merging tacrine and 3,4-dihydropyrimidin-2(1H)-thiones. Concerning the biological profile of tacripyrimidines, compound X12 appeared as the well-balanced cholinesterase inhibitor and calcium channel blocker, with an IC_50_ value of 3.05 *μ*M against hAChE and 3.19 *μ*M against hBChE. This compound had modest calcium channel blocking activity of 30.4% at 1 *μ*M; further, it did not cause any toxicity to HepG2 cells. X13 inhibits hAChE 10 times more effectively than tacrine, the strongest AChEI in the tacripyrimidine family. On the basis of hBuChE inhibition, X14 was the most effective with an IC50 of 0.372 *μ*M. In the test of Ca2^+^ channel blocking activity in SH-SY5Y neuroblastoma cells, X15 is the most strong, inhibiting 59.01%, followed by X16, which inhibited 66.79%, and the reference compound, nimodipine, which inhibited 49.62% [73].

Vilazodone and tacrine were combined to create a novel class of hybrid molecules for the treatment of depression associated with Alzheimer's disease. Additionally, vilazodone is an inhibitor of 5-HT reuptake and a 5-HT1A receptor partial agonist. Serotonin deficiency has been linked to a number of mental diseases, including depression. Compound IC50 values for acetylcholinesterase inhibition (3.319 M), 5-HT reuptake inhibition (76.3 nM), and 5-HT1A agonist (EC50 = 107 nM) were all within a reasonable range for 3-(4-(4-(4-(2,3-dihydro-1H-cyclopenta[b]quinolin-9-yl)piperazin-1-yl)butyl). It also significantly reduced hERG activity, exhibited tolerable hepatotoxicity, and could pass the blood-brain barrier in vivo. Cycle length has an effect on activity, which can be seen when the cycle size reduces: cycloheptane ring >cyclohexane ring >cyclopentane ring >no ring. The 5-HT1A antagonist's action increases as the cycle length lowers. Adding the naphthenic ring to 5-HT reuptake inhibitory activity resulted in a significant increase in potency. 3 (Butyl)-1H indole-5 carbonitrile-HCl was found to considerably boost cognitive function and reduce depressive features in mice [109].

Among all benzochromenoquinolinones, 14-amino-13-(3-nitrophenyl)-3,4-dihydro-1H-benzo[6,7]chromeno[2,3-b]quinoline-7,12(2H,13H)-dione had the most suppressive activities on the cholinesterase enzyme. This chemical inhibited AChE with an IC_50_ of 0.86 *μ*M and BChE with IC_50_ 6.03 *μ*M. Additionally, the IC_50_ value of this compound for BACE1 inhibition was found to be 19.60 *μ*M. It is capable of binding to both the PAS and CAS sites found on AChE and BChE. Considering they possess the suitable biological attributes and have a minimal hepatotoxicity, benzochromenoquinolinones have evolved as a flexible anti-AD drug [110].

A chloro substitution of tacrine and several of its analogues has been effective in managing the ChEI action in the hunt for powerful AChEI with minimal hepatotoxicity for the cure of Alzheimer's disease. Adding chlorine atom to tacrine's phenyl ring generated chlorinated tetrahydroacridines (THA) with considerable AChE inhibition. Furthermore, 3-cyano-2-substituted tetrahydroquinolines bearing a phenyl ring at the 4-position are also notable chlorinated analogues. When the 2-position of the pyridine ring had a chlorine atom added to it, this indicated the compound to be more efficacious than tacrine in inhibiting AChEI while also being less harmful to the liver. The chlorinated 4-phenyltetrahydroquinolines have improved AChE inhibitory potential and minimal hepatotoxicity compared with the unchlorinated analogues. In addition to this, compound 4-(pchlorophenyl)pyrazolo[3,4-b]tetrahydroquinoline showed encouraging activity, and its formation requires joining the pyridine ring with the pyrazolo nucleus. In addition, this compound was depicted to being completely risk-free [111].

Within the central nervous systems of mammals, glutamate is one of the most prominent excitatory neurotransmitters. Glutamate excitotoxicity, which is accountable for neurodegeneration in ischemic and traumatic brain damage, is triggered by an overstimulation of postsynaptic glutamate receptors, specifically NMDA subtype. Evidence shows that mitochondrial dysfunction is a key and initial mechanism in glutamate excitotoxicity. In terms of reducing glutamate-induced excitotoxicity, posttreatment with compound (17) is much more effective than posttreatment with NG-monomethyl-L-arginine. Compound (17) posttreatment may reduce glutamate excitotoxicity by blocking signaling pathways of mitochondrion apoptosis, while preserving proper mitochondrial performance. Besides these qualities, compound (17) suppresses glutamate-induced oxidative stress and NOS activity. Thus, this tacrine homodimer might pave the way for a new approach for curing neurodegenerative disorders [112].

In terms of hindering DMPP-mediated Ca^2+^ uptake, the novel tacrine derivatives were quite far highly effective and powerful. Compounds X19 and ethyl 5-amino-6,7,8,9-tetrahydro-4-(m-methoxyphenyl)-2-methylpyrano[2,3-b] quinoline-3-carboxylate were the most efficient blockers, inhibiting 90% and 88.5% of DMPP-stimulated Ca^2+^ entry, respectively. On the basis of animal studies, compound X20 has the most effective Ca^2+^-blocking efficacy (IC_50_ 0.3 *μ*M) in comparison to the commonly used L-type Ca^2+^ channel blocker, diltiazem (IC_50_ 0.03 *μ*M) [113].

Besides, tacrine forms hybrid molecules other many other compounds, such as 7-hydroxycoumarin, ferulic acid, acridine, cinnamic acid, phenolic acid, hydroxyphenyl benzimidazole, tryptophan, and indoles, which possess potential therapeutic activity against Alzheimer's disease (Table 1).

### 5.2. Parkinson's Disease

Parkinson's disease is caused by a decrease in the number of dopaminergic neurons in the substantia nigra portion of the brain. The presence and buildup of alpha-synuclein protein are the key pathological characteristic of Parkinson's disease. Due to the excessive buildup, levels of dopamine begin to decline over time, leading to a range of motor and nonmotor disorder. It can be treated but not cured completely. For the purpose of treating Parkinson's disease, a number of multifunctional targeted 3-arylcoumarin-tetracyclic tacrine derivatives were developed. Compounds X17 and X18 were shown to reduce synuclein protein aggregation as well as having antioxidant properties. These two compounds were found to be fascinating. In the case of Parkinson's disease, they also elevated levels of dopamine, which are essential and advantageous for relaying nerve impulses [133].

**Figure 4:**
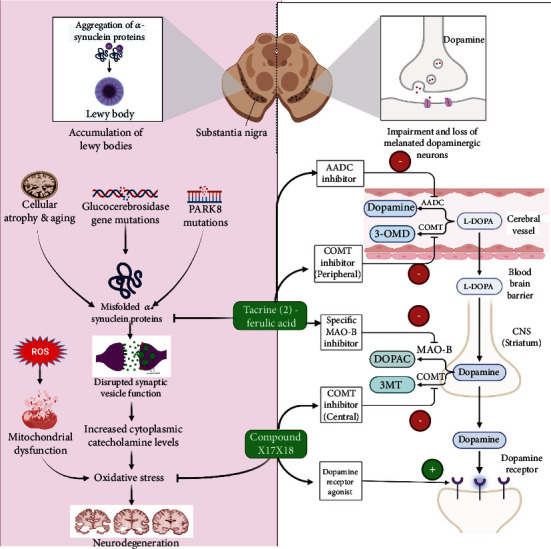
Illustration representing the site of action of different tacrine derivatives in Parkinson's disease.

Tacrine is used to induce tremulous jaw movements, a characteristic feature of Parkinson's disease in rat models to test the effect of anti-Parkinsonian agents [134–138]. Parkinson's disease cannot be treated with tacrine alone. 6-Hydroxydopamine (6-OHDA) is a neurotoxin used as a tool to induce Parkinson's disease in PC12 cells and rat models. 6-OHDA-induced Parkinson's disease in PC-12 cells can be lessened by the dimer of tacrine (2)-ferulic acid, which has been proven to reduce the symptoms of the condition. TnFA, *n* = 2 − 7, acetylcholinesterase inhibitory tacrine–ferulic acid dimers connected by an alkylenediamine side chain, were produced to test their effect on 6-OHDA-affected PC12 cells, according to a research conducted in 2011. At high concentrations and over a long period of time, 6-OHDA caused cell death in PC12 cells. T2FA (20 M) provided neuroprotection against 6-OHDA-induced cell death (69.35 3.69 percent survival, compared with control, *p* 0.01), whereas TnFA (*n* = 3–7) did not provide any protection. The authors also mentioned that ferulic acid or tacrine alone could not prevent 6-OHDA-induced cell death in the test subject. So, the protective effect is not caused by any monomer but the T2FA dimer (Figure 4). In addition, 20 M T2FA lowered the frequency of cells with apoptotic bodies and nuclear condensation, decreased intracellular ROS, blocked GSK3b activation, and reversed the inhibition of the PI3-K/Akt pathway produced by 6-OHDA. Researchers found that T2FA, an AChE inhibitor, reduced 6-OHDA-induced apoptosis in PC12 cells and so might be used to change the etiology of Parkinson's disease (PD) [57].

### 5.3. Ischemia

Tacrine has no therapeutic activity against cerebral ischemia. But a dimer of tacrine, compound (17) and has shown notable activities against cerebral ischemia according to a few studies. Comparable to memantine, a well-known NMDA receptor antagonist used to treat Alzheimer's disease, compound (17) has promising properties on blocking the NMDA receptor. For this reason, the ability of compound (17) to impede focal cerebral ischemic injury in rats with middle cerebral artery occlusion was examined. The results showed that compound (17) (0.1-0.2 mg/kg) could notably decrease the neurological problems and improve the neurological score and reduced infarction and brain edema after 2-h occlusion/2411 reperfusion. The neuroprotective activity of compound (17) was almost 260 times higher than that of memantine. So compound (17) could be useful as a neuroprotective agent for treating stroke as it can reduce neurological consequences of ischemic injury by NMDA receptor inhibition [139].

In another experiment, the influence of compound (17) on apoptosis caused by ischemia in astrocytes of mice cerebral cortex was examined. The astrocyte cell cultures were incubated in ischemic condition for 6 hours, and lactate dehydrogenase (LDH) release assay showed significant reduction in viable cells' percentage. Also, bisbenzimide staining determined that the anaerobic chamber caused apoptosis of 65% of the cells. During the ischemic incubation, the treatment with 1-10 nM concentration of compound (17) prevented this apoptosis of cells caused by oxygen deficiency, which was confirmed by both biochemical and morphological tests. So this drug can be beneficial against vascular dementia, which is caused by ischemia-induced death of the astrocytes [140].

Sprague-Dawley rats were used in another investigation to test the in vivo effects of compound (17) against localized cerebral ischemic injury. There was ischemia in the brain for 2 hours and 24 hours, and compound (17) was administered intraperitoneally after 15 minutes of this time period. Depending on the dose, the neurological abnormalities were improved, and both edema and cerebral infarct volume were decreased. It was shown is TUNEL staining assay that compound (17) reduced apoptosis of the neurons of penumbral region. The therapeutic window of compound (17) lasted until 6 hours which was wider than a similar NMDA receptor blocker memantine. But it did not change the blood flow of cerebral regions or any physiological variables in any stage of the experiment. So it can be concluded that the dose- and time-dependent effects of compound (17) can give protection against acute focal cerebral ischemic injury, probably through its antiapoptotic activity during numerous stages of the ischemic cascade [141].

Shu et al. stated that compound (17) at the dose of 0.2 mg/kg shortened the escape latency of adult male Sprague-Dawley rats whose spatial learning ability was impaired by chronic cerebral ischemia. Compound (17) lowered the rate of neuronal apoptosis in CA1 region of the hippocampus and enhanced neurogenesis in that region, compared to the saline-treated rats, as shown by immunohistochemical assay. Thus, it was suggested that compound (17) inhibits apoptosis and promotes neurogenesis in 2VO rats to exert its neuroprotective effects [38].

The neuroprotective impact of compound (17) on ischemia retinal injury was substantial. Compound (17) further prevented the reduction of amplitude of a- and b-wave in electroretinography, caused by ischemia. Ischemic damage led to a rise in the protein quantities of p53, which is a tumor suppressor gene well-known for its ability to trigger apoptosis. In such scenario, compound (17) therapy lowered the protein's expression. The treatment with compound (17) offered protection from damages of acute focal cerebral ischemia. Furthermore, glutamate excitotoxicity in the extracellular space of the retina is hypothesized to be one of the processes underlying neuronal cell death in glaucoma. Compound (17) outperformed memantine in glutamate-induced neurotoxicity in rats by specifically inhibiting the NMDA-activated current. In addition, compound (17) has the ability to inhibit the formation of NO which is induced by glutamate. As a corollary, compound (17) may hold promise as a neuroprotectant therapeutic agent for the treatment of glaucoma [142].

## 6. Concluding Remarks

This study provided a concise summary of the existing understanding about tacrine analogues that have genuinely valuable therapeutic effects. This article reviews the literature in the fields of medicinal chemistry, pharmacology, and biology pertaining to the topic. The authors described NO-donor-tacrine hybrids that displayed hepatoprotective features, as well as fused with natural components that protect cells from oxidative damage, such as melatonin, hydroxyquinoline, or thioflavine. Additionally, addresses a broad range of hybrid compounds that were developed by researching into the pharmacophores of currently marketed drugs. Other examples of hybrid compounds that were developed by studying the pharmacophore of commercial medications have been described. Structured hybrid inhibitors of cholinesterases have effectiveness in inhibiting cholinesterases activity, as well as antioxidant capacity and the ability to prevent aggregation A*β* (*β*-amyloid). As a result, these compounds have the potential to operate as neuroprotective medications while also displaying increased biological activity. The currently targetable pathways have been outlined in detail in order to facilitate the development of neurotherapeutic drugs. The article provides a concise update as well as their viewpoint on actual and possibly druggable pharmacological targets for the illness of the central nervous system.

This review highlighted a number of innovative multitarget-directed tacrine analogues that have been developed to date and are categorized based on their biological target and chemical composition. Novel therapeutic prototype candidates have been uncovered, and several are already undergoing preclinical testing. It is the starting point for a radical shift in how we approach medication development. System biology may help medical scientists uncover novel chemical entities capable of comprehending and efficiently modifying this severe neurodegenerative condition, despite the fact that none of these MTDL medication candidates has reached the clinical stage.

## Figures and Tables

**Table 1:** Experimental findings on the use of tacrine derivatives in neurological disorders.

Types of compound	Type of study	Study models	Dose/concentration	Assay type	Findings/activity	References
Tacrine-benzoate (phenyl acetates or cinnamates) hybrid	*In-vitro*	AChE, from electric eel, BuChE, from equine serum	5.63 nM	Ellman method	Inhibit AChE with highest selectivity ratio against BuChE	[114]

7-MEOTA-donepezil-like hybrids	*In-vivo*	Male Wistar rats	25.6, 12.3, 5.7, 5.2 mg/kg	Water maze test, passive avoidance test	Significant effect of the swim order indicating maintenance of learning ability	[115]

Tacrine-ferulic acid hybrids	*In-vitro*	AChE BuChE	61.7 ± 5.2 106.9 ± 13.1	Spectrophotometric method	Good inhibitory activity to both AChE and BuChE, better selectivity for AChE compared with tacrine	[100]
	*In-vitro*	A*β* (1–42)	20 *μ*M	Thioflavin T-based fluorometric assay	Similar inhibitory activity as curcumin and ferulic acid	[100]

Ferulic acid-tacrine-melatonin hybrids (FATMHs)	*In-vitro*	SH-SY5Y cells	1 *μ*M and 3 *μ*M	Neuroprotection analyses	Significant neuroprotection was observed against all toxic insults assayed	[116]

Tacrine-trolox, tryptoline hybrids	*In-vitro*	TcAChE from electric eel, eqBuChE (from equine serum)	49.31 nM 17.37 nM	Ellman's assay	Hybrids with longer linker chain lengths show increased AChE inhibitory activities compared to the shorter ones	[117]

Tacrine-cinnamic acid hybrids	*In vivo*	Adult ICR mice	15 mg/kg	Morris water Maze test	Considerably ameliorated the cognitive impairment of the treated mice And was much better than tacrine	[118]
				ALT & AST level test	Did not show any hepatotoxicity at all the time points	[118]
	*In vitroIn vivo*	AChE, BuChE, A*β* (1–42) ICR mice	10.2 nM 6.3 nM 30 mg/kg	Ellman's Assay Thioflavin T-based fluorometric assay Morris water Maze test	Cholinesterase inhibitory activities, amelioration of scopolamine-induced cognition impairment, preliminary safety in hepatotoxicity evaluation	[119]

(Benz)imidazopyridino tacrines	*In-vitro*	EeAChE eqBuChE	0.50 ± 0.03 *μ*M	Ellman protocol	Nonhepatotoxic shows moderate and selective EeAChE inhibition	[120]

Tacrine-O-protected phenolics heterodimers	*In-vitro*	AChE, from electric eel, BuChE, from equine serum	3.5 *μ*M	Ellman method	Safe, nonhepatotoxic, potent, and selective inhibitor of hBuChE	[121]

Tacrine-resveratrol-fused hybrids	*In-vitro*	AChE, from electric eel, BuChE, from equine serum	8.8 *μ*M	Ellman method	AChE inhibition, A*β* self-aggregation modulation, anti-inflammatory, and immunomodulatory properties, high-predicted blood-brain barrier permeability, and low cytotoxicity	[49]

Tacrine-ferulic acid hybrids	*In-vitro*	A*β* (1–42)	20 *μ*M	Thioflavin T-based fluorometric assay	Inhibited amyloid *β*-protein self-aggregation by 65.49%	[122]
	*In-vitro*	AChE, from electric eel, BuChE, from equine serum	37.02 nM	Ellman method	Potent inhibitor against AChE and strong inhibitor against BuChE	[122]
	*In vivo*	Adult ICR mice	30 mg/kg	Morris water maze test, serum ALT, AST test	Ameliorated the cognition impairment and showed preliminary safety in hepatotoxicity evaluation	[122]

Tacrine-acridine hybrids	*In-vitro*	AChE, from electric eel, BuChE, from equine serum	7.6 pM 1.7 pM	Ellman method	More active inhibitor than tacrine	[123]
	*In-vitro*	A*β* (1–42)	50 *μ*M	Thioflavin T (ThT) fluorescence assay	54.74% inhibition of A*β* aggregation	[123]

Tacrine-deferiprone hybrids	*In-vitro*	AChE, from electric eel	0.64 *μ*M	Ellman method	Control of cholinergic dysfunction, amyloid peptide aggregation, oxidative stress, and metal modulation	[124]

Tacrine, phenolic acid, and ligustrazine hybrids	*In-vitro*	AChE, from electric eel	3.9 nM	Ellman method	Potent inhibition activity towards cholinesterases (ChEs)	[125]
	*In-vitro*	—	85.8 ± 3.5 *μ*M	DPPH assay	Very potent peroxyl radical scavenging capacity	[125]

Cystamine-tacrine dimer	*In-vitro*	SH-SY5Y cell line	0.005-0.5 *μ*M	MTT assay Enzymatic assay Fluorometric assay	AChE and BChE inhibitor; activates kinase 1 and 2 (ERK1/2) and Akt/(PKB) pathways	[92]

Tacrine-trolox hybrid	*In-vivo*	Male Sprague-Dawley (SD) rats	6 mmol/100 g b. wt	(AST) and (ALT) activity	Introduction of trolox could reduce the hepatotoxicity of tacrine Inhibitor against AChE and BuChE	[93]
	*In-vitro*	Electric eel, Ellman's reagent, DTNB	9.8-23.5 nM 20.5-22.2 nM	Ellman's assay	More potent inhibitory activity for BuChE than for AChE	[93]
		PC12 cells	3.125 *μ*M and 6.25 *μ*M	MTT assay	Significantly inhibit cell death	[93]

Tacrine-propargylamine derivatives	*In-vitro*	Human neuroblastoma cell line, SH-SY5Y	10, 50, and 100 *μ*M	MTT assay	Nearly no effect on the viability of SH-SY5Y cells, lower cytotoxicity than tacrine	[126]

Tacrine-coumarin hybrids	*In-vitro*	hMAO-A, hMAO-B	0.24 *μ*M	Fluorimetric method	Selective MAO-B inhibitor	[94]
	*In-vitro*	eeAChE, hBuChE	16.11 ± 0.09 nM 112.72 ± 0.93nM	Ellman's method	Potent inhibitory action for AChE and BuChE	[94]
	*In-silico*	Recombinant hAChE	—	—	Simultaneously bind to PAS and CAS and the mid-gorge site of AChE	[94]
	*In-vitro*	hAChE, hBuChE	38 nM 63 nM	Ellman's method	Potent and selective inhibitory activities towards both hAChE and hBuChE	[127]
	*In-vitro*	A-*β*1-40 peptide	1 *μ*M	Thioflavin T assay	Inhibit A-*β*40 amyloid self-assembly	[127]
	*In-silico*	hAChE(1ACJ), hBuChE (4 BDS), *β*-secretase (BACE1)	—	AutoDock 4.2 and Vina	Fingerprints studies showed 34 ligands to be effective in their docking binding energies and high binding natures	[128]

Tacrine-benzofuran hybrids	*In-vitro*	Recombinant hAChE and hBChE	0.86 nM 1.35 *μ*M	Ellman's assay	Selectively inhibited hAChE, suppressed both hBACE-1 activity and *β*-amyloid aggregation	[95]
	*In-vivo*	ICR mice	20 *μ*mol/kg	Morris water maze test	Considerably ameliorated the cognition impairment of the treated mice	[95]

Tacrine/cysteine-conjugated compounds	*In-vitro*	Amyloid-*β* peptide	70 *μ*M	Fluorescence assay	Decreased A*β*_42_ (40 *μ*M) self-aggregation	[96]
	*In-vitro*	Human neuroblastoma SH-SY5Y cells	2.5 *μ*M	MTT assay	Cell viability is not significantly affected after a 24-h treatment	[96]
		TcAChE	0.30 *μ*M	Ellman0s assay	High inhibitory activity in submicromolar range	[96]
	*In-silico*	—	—	Molecular docking	Target both the CAS and PAS of AChE	[96]

Tacrine-phenylbenzothiazole hybrids	*In-vitro*	AChE from electric eel A*β* (1–42)	0.15 *μ*M 40 *μ*M	Modified method of Ellman's assay Thioflavin T (ThT) assay	Excellent AChE inhibitory activity and moderate inhibition values for amyloid-*β* (A*β*) self-aggregation (27–44.6%)	[129]

Tacrine-1,2,4-thiadiazole derivatives conjugates	*In-vitro*	Human erythrocytes AChE equine serum BChE, porcine liver CES	17.1 *μ*M 44.8 *μ*M	Ellman method	Effectively inhibited cholinesterases with a predominant effect on (BChE), could block AChE-induced *β*-amyloid aggregation	[130]

Tacrine-hydroxamate derivatives	*In-vitro*	AChE BChE HDAC	0.12 nM 361.52 nM 0.23 nM	Ellman method Fluorescence assay	Potent and selective inhibition on ache, potent inhibition on HDAC, recognitive impairments inhibitory activity on A*β*1-42 self-aggregation as well as disaggregation activity on preformed A*β* fibrils	[47]

Tacrine-pyrimidone hybrids	*In-vitro*	Murine AChE Recombinant human GSK-3*β*	51.1 nM 89.3 nM	Ellman's method with modification	Possessed excellent dual AChE/GSK-3 inhibition both in terms of potency and equilibrium	[48]
	*In-vivo*	Female ICR mice	15 mg/kg	Morris water maze (MWM) tests	Displayed significant amelioration on cognitive deficits in scopolamine-induced amnesia mice	[48]

Tacrine(10)-hupyridone dimer	*In-vivo*	Wild-type (WT) mice	0.36 or 0.72 *μ*mol/kg	Morris water maze (MWM) tests	Long-term treatment of the compound could attenuate precognitive impairments in APP/PS1 transgenic mice	[131]

Tacrine and salicylamide conjugates	*In vitro*	AChE BChE	0.22 *μ*M 0.01 *μ*M	Propidium iodide fluorescence	Exhibited high dual anticholinesterase activity with selectivity towards BChE	[132]

## Data Availability

All data used to support the findings of this study are included within the article.
